# The Surgeons of Baltimore and Their Achievements

**Published:** 1880-11

**Authors:** 


ARTICLE II.

### THE SURGEONS OF BALTIMORE AND THEIR ACHIEVEMENTS.

Read before the Medical and Chirurgical Faculty of Maryland, at a special meeting held Oct. 13th, 1880, for the purpose of taking part in the Sesqui-Centennial Celebration of Baltimore, and ordered to be published, in full, in their next (1881) Annual Transactions, and also in the Sesqui-Centennial History.

[We are indebted to the courtesy of Dr. B. Bernard Browne, the author, for the privilege of placing before our readers the below extracts from his valuable paper, prepared by appointment and read before the Medico-Chirurgical Faculty of Maryland. Further extracts will be made from this admirable epitome of the work of Baltimore Surgeons in the succeeding issue of the journal, and we feel assured in advance, that they will be read with interest by the profession at home and abroad.]

Dr. Browne said, now that we are celebrating the 150th Anniversary of the foundation of Baltimore, it seems appropriate that we should look back over these past years and see what part medical men and the medical profession have taken in its affairs and how far they have aided and contributed to its growth and prosperity. From the earliest settlement of the State under the Proprietary Government, the medical men who emigrated here, had for the most part received their education either at London, Edinburg or Glasgow, and we find them taking an active and prominent part in the affairs of the colony.

When the act was passed in 1729, entitled "An Act for erecting a town on the north side of the Patapsco, in Baltimore county, and for aying out into lots, 60 acres of land," &c., we find that Dr. George Walker and Dr. George Buchanan were appointed Commissioners and aided in laying out the Town on January 12th, 1730.

Dr. Walker had previously practiced medicine in Anne Arundel county for some years, and came to reside in Baltimore county about 1715, he was the proprietor of a well known seat and tract of land on the west side of the town, called Chatsworth.

Dr. Buchanan who came from Scotland, purchased lands and practised medicine in the county from the year 1723 until his death in 1750.

About 1760, Dr. Henry Stevenson, from Ireland, settled in Baltimore and engaged in the practice of medicine and surgery. He built a large stone house near the York road, and in 1769 he converted this new and elegant residence, which on that account was called " Stevenson's Folly," to the very laudable purpose of a small-pox hospital, by appropriating part of it for the reception of young men whom he inoculated successfully before the practice became general.

Although this paper was designed to record the achievements of Baltimore Surgeons, there was no separate and distinct class of such persons at that time, and even now there is not a medical man in the city who devotes himself exclusively to surgical practice, and there were but few physicians who did not treat the more common surgical diseases and injuries.

As there was no medical journal published in America previous to the " Medical Repository," which was first issued in 1797, it has been impossible to get the authentic record of any surgical operations of importance which were performed up to that time.*

In the autumn of 1796, Dr. John Beale Davidge, a native of Annapolis, who had been educated in Europe, and graduated at Glasgow,† settled in Baltimore. He was the founder of the University of Maryland, in which he occupied for many years the chairs of Anatomy and Surgery. He enjoyed a high reputation as a teacher and surgeon. He was the first Surgeon in this country who tied the gluteal artery for the cure of aneurism ; his patient recovered although he lost much blood from hemorrhage.

In April, 1823, Dr. Davidge tied the carotid artery, for a fungus growth in the antrum of the left side of the face, about 5 weeks after the operation, the patient was free of complaint and breathed with ease and comfort, but upon being removed to his home he was at-

*The first medical man of whom there is any specal reference as a Surgeon was Dr. Chas. Frederick Wiesenthall, who was Surgeon to Col. Wm. Smallwood's Battalion in 1776. And in 1789 when the first attempt was made to establish a medical school here, his son Andrew Wiesenthall, delivered a course of lectures on Anatomy, and Dr. Lyde Goodwin on the Theory and practice of Surgery.

The site of the first hospital established in Baltimore was selected by Captain Yellott and others, as a temporary retreat for the strangers and sea-faring people during the epidemic of yellow fever which raged here in 1794. In 1798 it was purchased by the city, and in 1808 it was leased to Drs. James Smythe and Colin MacKenzie, who conducted it as a general hospital. It afterwards became the Maryland Hospital for the Insane and is now occupied by the Johns Hopkins Hospital.

†His thesis, written in Latin, and first printed in Glasgow, in 1793, maintains the doctrine that the menstrual flow is a true secretion.

tacked with tetanus, and died about six weeks after the operation.

In reporting the case, Dr. Davidge remarks, "It may be of moment that I mention what attentions were directed to the tumor, subsequently to the tying of the artery. The tumor was left to itself, protruded down through the opening in the bone. It gradually fell into mortification, and sloughed away so completely that no vestige could be discovered; no part was removed by knife, scissors or caustic." Dr. Davidge was the originator of the "American plan of amputation."

In September, 1805, Drs. Colin MacKenzie and James Smythe succeeded in reducing a laxated humerus, which had been out of place more than 5 months. Dr. John Syng Dorsey in his Surgery speaks of this as a truly remarkable case, of the success of which he believed no parallel is to be found in the records of Surgery, and that Dr. MacKenzie had accomplished what no other surgeon of this or former ages can boast of having performed. During the same year Prof. James Cocke of Baltimore, also reduced successfully a dislocated shoulder of four months standing.

Dr. William Gibson was born in Baltimore in 1784, and after receiving a classical education went abroad. He took his medical degree in Edinburg, and through the friendship of Sir Charles Bell, obtained extraordinary advantages there. He was the first surgeon that ever ligated the common iliac artery, an operation that contributed greatly to his reputation; this he did during the riots in Baltimore in 1812, for the arrest of hemorrhage caused by a gun-shot wound of the abdomen; two convolutions of the intestines were wounded, each opening was closed with a ligature and returned. He was the first surgeon in this country to perform the supra-pubic operation of lithotomy.

In 1828, he ligated the subclavian artery in its third surgical division, for hemorrhage from the axillary artery which was wounded in the reduction of the shoulder joint.

He also invented an apparatus for the treatment of fractures of the lower jaw, which held a high place in the esteem of American surgeons, and was far in advance in point of simplicity and efficiency of those used by European surgeons.

He performed the Cæsarian operation twice upon the same patient, saving each time the mother and child.

In the American appendix to Cooper's Dictionary of Surgery, issued in 1842, the editor states that the straight muscles of the eye were divided by Prof. Wm. Gibson, for the care of strabismus several years before the operation was performed by Dieffenbach of Berlin. Dr. Gibson occupied the chair of Surgery in the University of Maryland, from 1813 to 1818, when upon the death of Prof. Dorsey, his professional influence was so great that he secured the chair of Surgery in the University of Pennsylvania, which was then occupied by Dr. Physick, the Father of American Surgery, who consented to take the chair of Anatomy with an adjunct.

Dr. Gibson occupied the chair of Surgery in the University of Pennsylvania from 1818 to 1854. He was the author of a work entitled "The Institutes and Practice of Surgery."

He was a cotemporary of Physick, Warren, Mott and Dudley, names intimately connected with the rise and progress of Surgery in America.

Dr. Gross speaks of him as an accomplished lecturer, a lucid writer and an able speaker.

After the removal of Dr. Gibson to Philadelphia, Dr. Granville Sharp Pattison, who had then lately arrived from Scotland, was elected in 1820, professor of Surgery in the University of Maryland, he was a pupil of Allan Burns of Glasgow, the author of the great work "Observations on the Surgical Anatomy of the Head and Neck," of this work Dr. Pattison brought out an edition in this country.

After retiring from the University of Maryland, he occupied successively the chair of Anatomy in the London University, in the Jefferson Medical College and in the University of the City of New York. He was one of the ablest teachers of surgical anatomy of the age, and by his enthusiasm as a lecturer he had the happy faculty of inspiring his pupils with a love for surgery such as few men ever possessed.

In April, 1821, he made a partial excision of the superior maxilla for a tumor of the antrum, which he cut away and chiseled and malleted off much of the bones of the face and took out the eye.

Horatio Gates Jameson, Surgeon to the Baltimore Hospital, was no doubt one of the ablest surgeons of his day. In referring to his excision of the superior maxilla, Dr. Gross says: "America may justly claim the honor of having led the way in extirpations of the *upper* jaw, Small portions, it is true, had been chipped off in the 18th, and even the 17th century; but the first grand and difficult operation of the kind of which we have any knowledge, was performed in 1820, by Horatio G. Jameson, of Baltimore, who took away nearly the entire bone on one side, the roof of the antrum alone being left, as it was not involved in the disease. As a preliminary step to the operation the carotid artery was ligated, both to prevent hemorrhage and to cut off the future supply of blood. The operation was successful and the patient recovered.

In May, 1821, he ligated the external iliac artery for the cure of aneurism.

On May 14th, 1823, he performed tracheotomy successfully for the removal of a pebble from the trachea. This was probably the first time the operation was done in Maryland.

In 1824 he published an essay on Stricture of the urethra and its treatment by dilatation, with a report of cases.

He also published an essay on the "Surgical Anatomy of the Neck," and another on the "Surgical Anatomy of the parts concerned in the operation of tying the arteria innominata, illustrated with transverse sections.

In January, 1827, his Prize Essay "Observations upon Traumatic Hemorrhage, illustrated by experiments upon living animals," was published in the *Medical Recorder*. He also wrote able and exhaustive articles on Lithotomy, Hernia, Fistula in Ano, Stricture of the Rectum, Aneurism, Yellow Fever, and many other subjects.

In May, 1824, he amputated the cervix uteri for scirrhous, which was the first time that this operation was done in Great Britain or America. Prof. Osiander of Gottingen having previously operated in 1802, and Lisfranc in 1816. The manner in which he prepared himself for this operation shows that he was a surgeon at least fifty years ahead of the times in which he lived. He provided himself with forceps which had deep teeth on the outside of their beaks, and made to stand open by means of a screw through their handles. It was intended that the beaks when closed should be passed into the uterine cavity, and then on opening them, they would serve to bring down the uterus.

Dr. Jameson was the first surgeon in Baltimore to attempt the operation of ovariotomy which he did previous to 1824, but unfortunately, the patient was so feeble that he had to desist.

A remarkable feature in the career of this surgeon was a medico-legal suit instituted it appears, by his rivals in surgical practice, who were determined to crush him out of existence. The prosecuting witnesses against him were some of the most prominent surgeons of his day, their testimony was bitter and biased by prejudice, and such was the zeal of one of them, that the chief judge asked him aloud, whether he was employed to plead the cause. These principal witnesses in cases they had not seen were confronted by disinterested men, who were at least their professional equals, and who said that Dr. Jameson had performed these operations not only in their presence but with their approbation, and in every case it was said that he acquitted himself well as an operator.

The case of amputation of the cervix was one of those for which he was accused of mal-practice. In his testimony one of the witnesses said that "he had examined this woman previously by the touch, that he never used the speculum uteri, which was proved to have been used by Dr. Jameson, the reason he never used it was *that it smacked of something unprofessional*, and upon being asked of what it did *smack*, he replied, "*Immoral* curiosity."

From the time of Jameson's attempted ovariotomy until within the past few years any Baltimore surgeon who performed the operation did it at the risk of his professional reputation.

